# Digital health in dermatology

**DOI:** 10.1016/j.jdin.2023.08.008

**Published:** 2023-08-20

**Authors:** Jonathan Kantor

**Affiliations:** Department of Dermatology, Center for Global Health, and Center for Clinical Epidemiology and Biostatistics, Perelman School of Medicine at the University of Pennsylvania, Philadelphia, Pennsylvania; Florida Center for Dermatology, St Augustine, Florida; and Alchemy Labs, Oxford, UK

**Keywords:** artificial intelligence, ChatGPT, copilot, digital health, Google Bard, large language models, machine learning, medical education, Microsoft Bing, practice management, teledermatology, teledermoscopy, telemedicine, whole slide imaging

Digital health in dermatology is at a crossroads. For years, the promise of transformative change has been heralded, and for years a range of stakeholders—dermatologists, trainees, pharmaceutical and biotech companies, and the general public–have been awaiting with near-messianic fervor (and patience) the immanentization of the digital revolution. Yet, until recently, beyond subtle improvements in teledermatology, dermatology has been dermatology as usual.

The warm-up phase has ended. With the rise of machine learning and artificial intelligence, and with the now difficult-to-ignore fluency of ChatGPT and other large language models, the era of digital dermatology is upon us. This virtual special issue of *JAAD International* highlights only the tip of the digital iceberg, with a wide range of articles on the usual suspects such as teledermatology,^1^^,^^2^ and some focusing on the next big things: teledermoscopy,^3^ machine learning and artificial intelligence,^4^ and wearable devices.^5^

These are not subtle developments, and they will have transformative effects on education, clinical care, and dermatopathology. Indeed, as automated whole slide imaging becomes more mainstream, relying on artificial intelligence systems as a copilot will become de rigueur. Although this may have a moderating effect—making less astute dermatopathologists a bit better—there is also the risk that we could come to lean too heavily on our electronic crutches, developing a generation lacking some of the fundamental skills that define us as physicians. As anyone who has used GPS assistance in a new city (and is old enough to recall navigating without its help) can attest, there is indeed a fundamental shift in skills and attention when a ubiquitous tool is broadly available. Indeed, the rising stress on imaging in medical care in lieu of the physical examination is likely a harbinger of things to come, and the ethical challenges latent in a future reliance on digital tools are too numerous to count.

ChatGPT and large language models, despite their banal platitudinous style, will clearly have a transformative effect in dermatology and beyond. The next step, applying ChatGPT-level fluency to image analysis, is arriving now, and in dermatology (as in radiology), this will prove to have far-reaching consequences. Those who ignore the past are doomed to repeat it. Those who ignore the future may not fare all that well either.

## Conflicts of interest

None disclosed.
